# Effectiveness of a video-feedback intervention based on the SPIRE happiness model for promoting family parenting among preschool migrant children: a pilot randomized controlled trial

**DOI:** 10.3389/fpsyg.2026.1915668

**Published:** 2026-09-11

**Authors:** Chenchen Yang, Mengxue Zhang

**Affiliations:** College of Educational Sciences, Nantong University, Nantong, China

**Keywords:** family parenting environment, parent–child interaction, pilot randomized controlled trial, preschool migrant children, video-feedback intervention

## Abstract

**Introduction:**

Preschool migrant children may face multiple family-related developmental risks, including suboptimal parent–child interaction. This pilot randomized controlled trial examined the feasibility, acceptability, and preliminary outcomes of a video-feedback intervention (VFI) informed by the SPIRE happiness model among Chinese families with preschool migrant children.

**Methods:**

A single-blind, parallel-group, pilot randomized controlled trial was conducted. Parents of preschool migrant children were recruited from kindergartens included in the migrant child sample database of Nantong City in September 2025 and were randomly assigned to either an intervention group (*n* = 14) or a control group (*n* = 13). The intervention group received a six-week video-feedback intervention, whereas the control group received no intervention. Assessments were conducted at pre-intervention (T1), immediately after the intervention (T2), and at the three-month follow-up (T3). Primary outcomes were feasibility, acceptability, and parent–child interaction behaviors; secondary outcomes were the family parenting environment, parenting sense of competence, perceived social support, and child emotional and behavioral problems.

**Results:**

A total of 27 participants completed the study with 100% retention and intervention attendance rates. Intervention satisfaction was 92.9, and 85.8% of parents were willing to apply the learned strategies and participate in similar interventions. Repeated-measures ANOVA demonstrated that following the intervention, the intervention group scored significantly higher than the control group on parent–child interaction, family parenting environment, parental sense of competence, and perceived social support (*p* < 0.05), and these improvements could still be observed at the 3-month follow-up. Although the scores of children’s emotional and behavioral problems exhibited a certain decreasing trend, the between-group difference was not statistically significant (*p* > 0.05).

**Discussion:**

These findings suggest that the VFI based on the SPIRE happiness model was feasible and acceptable in this sample and was associated with preliminary improvements in improving the quality of parent–child interaction, optimizing the family parenting environment, and enhancing parents’ positive psychological resources. Larger, adequately powered trials with longer follow-up periods are needed to evaluate its effectiveness and underlying processes.

## 1 Introduction

The preschool years, typically spanning ages 3–6, represent a formative period for children’s physical and psychological development and social adjustment. High-quality parent–child interactions and a supportive family parenting environment provide an important foundation for early development (Kohlhoff et al., 2022; Fazrin, 2023) and are also associated with subsequent mental health and social adjustment (Bechtiger et al., 2022). As urbanization continues in China, the development and wellbeing of migrant children have attracted increasing attention. In China, “migrant children” refers to individuals under 18 years of age who have relocated with their parents from rural to urban areas for at least 6 months without obtaining urban household registration (National Bureau of Statistics, 2021; Zheng and Zhou, 2024). According to the latest census data (2021), the total number of migrant children in China stands at 71.09 million, of which 19.55 million are preschool migrant children aged 0–5 years. The parenting and developmental support needs of this population therefore warrant close attention. Research suggests that, compared with their non-migrant peers, migrant children may face greater challenges in cognitive development, social adjustment, and mental health (Boelens et al., 2020). Migration per se, however, may not constitute a direct developmental risk. Rather, children’s development may be shaped by the accumulation of migration-related disadvantages, including less supportive family parenting environments, limited social support, and unequal access to educational resources (Evans et al., 2013; Medaric et al., 2022; Sun et al., 2025). Accordingly, family-based early intervention aimed at strengthening parenting conditions and supporting positive parenting practices has become an important direction for promoting the early development of migrant children.

Within family-centered early parenting interventions, video-feedback intervention (VFI) emphasizes observation and positive feedback grounded in actual parent–child interactions and has been widely used to improve parent–child interaction and support positive parenting practices (Montirosso et al., 2020; Favieri et al., 2026). However, existing VFI research has focused primarily on observable interaction behaviors and parenting practices, with comparatively less attention to the family and psychological factors that may help sustain positive parenting over time (Wan et al., 2025). For resource-constrained families of preschool migrant children, interventions focused only on parenting behavior may be insufficient to support the continued implementation of positive parenting practices (Nomaguchi and Milkie, 2020; Al Sager et al., 2024). Greater attention to parents’ positive psychological resources and relational experiences may therefore extend the theoretical scope and practical value of VFI. The present study integrated the SPIRE happiness model with VFI and developed an intervention tailored to Chinese families of preschool migrant children to evaluate its feasibility, acceptability, and potential preliminary outcomes.

## 2 Literature review

### 2.1 Parenting challenges and the need for early family-based intervention in families of preschool migrant children

Family systems theory conceptualizes children’s development as embedded within an interconnected and dynamic family relational system (Gavazzi and Lim, 2023). From this perspective, family members and relational processes are interdependent rather than functioning in isolation. Patterns of parent–child interaction, the emotional climate of the family, parents’ psychological functioning, and available family resources are mutually linked and jointly shape children’s developmental environments (Lindstedt et al., 2024). In migrant families, chronic stressors such as frequent relocation, resource constraints, and limited social support may increase parenting stress, weaken parents’ sense of competence, and compromise the quality of parent–child interactions and the family parenting environment (Puff and Renk, 2014; Dominguez et al., 2025; Karwatowska et al., 2025; Zhang et al., 2025). Together, these family-level conditions constitute important features of children’s early developmental environments and suggest that parenting processes may be a key target for supporting migrant children’s development.

From a family systems perspective, strengthening parenting processes represents an important point of intervention for supporting the early development of migrant children. A growing body of evidence suggests that family-based early interventions can support children’s longer-term development by strengthening parenting practices, improving parent–child interaction quality, and enhancing the family parenting environment (Provenzi et al., 2020; Sprenger et al., 2025). Nevertheless, evidence-based interventions specifically developed for families of preschool migrant children remain limited, and some families may have difficulty obtaining sustained support because of restricted access to services and limited opportunities for participation (Mansoor et al., 2025). These gaps underscore the need for feasible family-based interventions that can support positive parenting practices under resource-constrained conditions.

### 2.2 Video-feedback intervention: evidence and limitations

In recent years, family-centered early childhood interventions have increasingly shifted away from didactic parent education toward approaches that promote parental observation, reflection, and positive interaction. VFI has become a widely used strategy within this broader shift. Grounded in a strengths-based perspective, VFI involves reviewing recordings of actual parent–child interactions to help parents recognize children’s behavioral cues, reflect on their interaction patterns, and reinforce positive parenting behaviors (Montirosso et al., 2020). Because it is embedded in real family interactions, VFI is typically brief, encourages active parental engagement, and facilitates the transfer of intervention strategies to everyday family settings. These features may make it particularly suitable for resource-constrained families (Klein Velderman et al., 2006). Randomized controlled trials and systematic reviews indicate that VFI can enhance parental sensitivity and positive parenting and may promote children’s attachment security. However, evidence regarding its effects on parenting stress, parenting sense of competence, child behavioral problems, and broader family functioning remains mixed (O’Hara et al., 2019; van IJzendoorn et al., 2023). The effects of VFI may vary according to caregivers’ psychological status, intervention dose, and mode of delivery (Favieri et al., 2026). Moreover, a qualitative meta-synthesis found that existing VFI research has focused primarily on behavioral changes in parent–child interaction, while giving comparatively less attention to parents’ psychological resources, overall family wellbeing, and the processes through which intervention gains are maintained (Wan et al., 2025). Chinese families of preschool migrant children may face persistent challenges during urban adaptation, including limited time for parenting and educational involvement, difficulties accessing educational resources, inadequate social support, and elevated parenting stress (Liu, 2025). Addressing parents’ positive psychological resources and the supportive family context, alongside changes in parenting behavior, may therefore help sustain positive parenting practices.

### 2.3 Theoretical rationale for integrating the SPIRE happiness model with VFI

The SPIRE happiness model, proposed by Ben-Shahar (2021), conceptualizes wellbeing as a dynamic process involving five interrelated dimensions: Spiritual, Physical, Intellectual, Relational, and Emotional wellbeing. The model proposes that wellbeing depends not only on positive emotional experiences but also on meaning-making, continued learning, interpersonal connectedness, and physical and psychological health (Ben-Shahar, 2021). Recent research in positive psychology further suggests that parents’ sense of meaning in parenting, positive emotions, reflective capacity, and social support are important not only for their own wellbeing but also for positive parenting and the maintenance of family functioning (Nomaguchi and Milkie, 2020; Hoang et al., 2024; Antolín-Suárez et al., 2026; Nomaguchi and Milkie, 2017). The SPIRE happiness model may therefore offer a positive psychology framework for addressing both behavioral and psychological change within VFI. Rather than simply adding psychological support to behavioral training, the integrated approach embeds attention to parents’ positive psychological resources and relational experiences within the video-feedback process. It enables parents not only to observe and adjust their interaction behaviors but also to understand and refine their parenting practices in relation to parenting meaning, emotional experience, and relational connectedness. Such an approach may extend VFI beyond its conventional emphasis on behavioral change and provide a more comprehensive theoretical basis for developing and sustaining positive parenting practices.

### 2.4 The present study and hypotheses

Guided by this framework, the present study developed an integrated VFI program informed by the SPIRE happiness model and evaluated it in a pilot randomized controlled trial. While retaining VFI’s core emphasis on improving parent–child interaction, the integrated program also addressed parents’ positive psychological resources and broader parenting processes within the family. The program was designed to facilitate the application of positive interaction strategies in everyday family life and to explore its potential effects on the family parenting environment, parental psychological resources, and child-related outcomes.

Because feasibility and acceptability are usually central outcomes in pilot intervention research (Klein et al., 2021), and parent–child interaction is a key proximal outcome of VFI (Juffer et al., 2017), the primary outcomes were intervention feasibility, acceptability, and preliminary changes in parent–child interaction behaviors. Secondary exploratory outcomes included the family parenting environment, parenting sense of competence, perceived social support, and child emotional and behavioral problems. Accordingly, three hypotheses were proposed. H1: The intervention would be feasible and acceptable for Chinese families of preschool migrant children. H2: Compared with the control group, the intervention group would show more favorable changes in parent–child interaction behaviors. H3: Compared with the control group, the intervention group would show more favorable changes in the family parenting environment, parenting sense of competence, and perceived social support, while child emotional and behavioral problems would show a favorable trend.

## 3 Materials and methods

### 3.1 Study design and procedure

**Figure 1:**
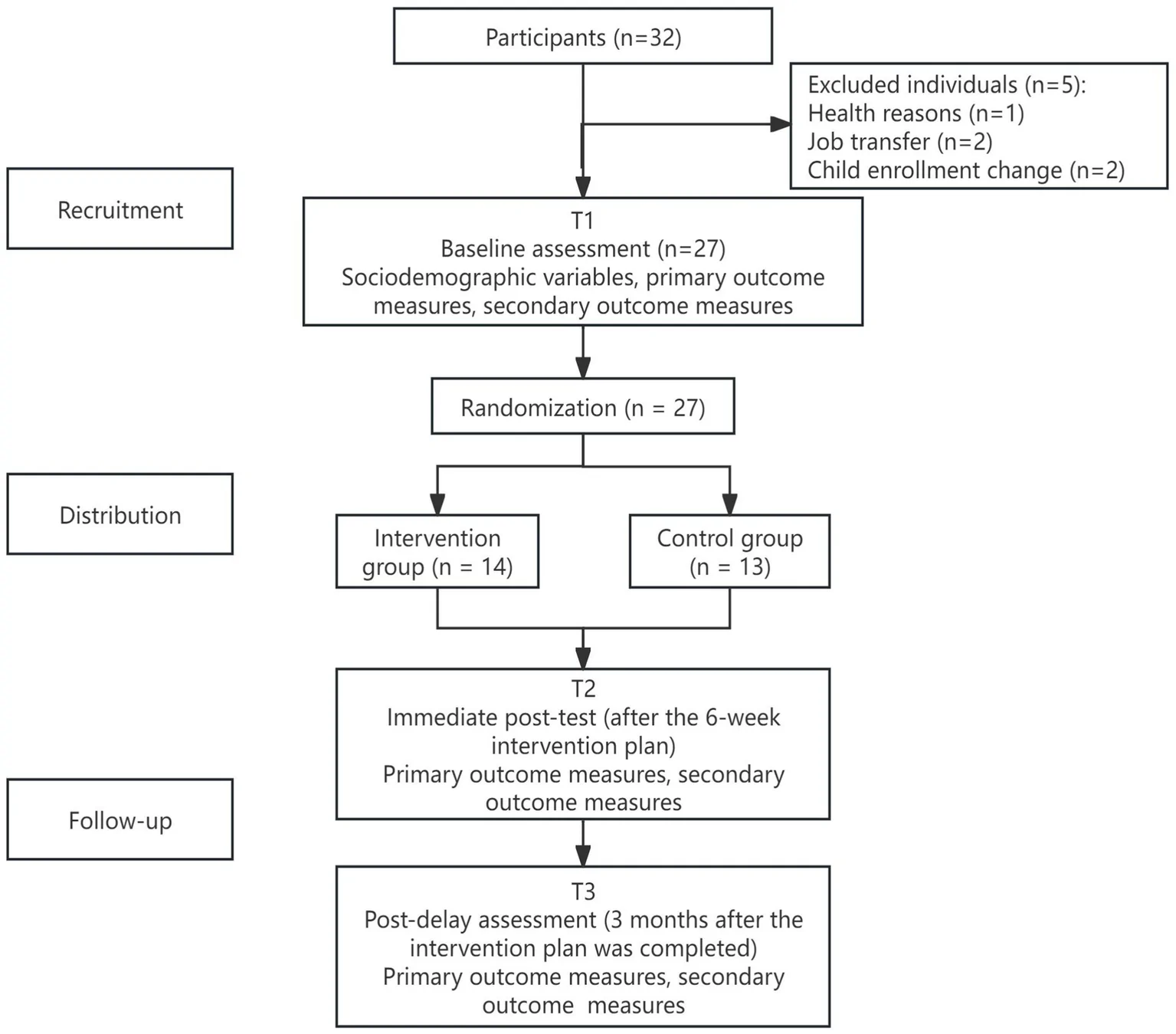
Research procedure of the video-feedback intervention (VFI).

This study was a single-blind, parallel-group pilot randomized controlled trial with three assessment points (Figure 1): baseline before the intervention (T1), immediately after the 6-week intervention (T2), and 3 months after the intervention ended (T3). After eligibility was confirmed, written informed consent was obtained, and baseline assessments were completed, participants were randomly allocated to the intervention or control group in a 1:1 ratio. Randomization was conducted by an independent researcher who was not involved in participant recruitment, intervention delivery, outcome assessment, or data analysis. The allocation sequence was generated using computer-generated random numbers, and group assignments were placed in sequentially numbered, opaque, sealed envelopes to ensure allocation concealment. Because participants and intervention providers could not feasibly be blinded in this behavioral intervention, a single-blind design was used. Researchers responsible for outcome assessment, assessment using the Keys to Interactive Parenting Scale (KIPS), and data analysis remained blinded to group allocation to reduce the risk of assessment bias.

After randomization, participants in the intervention group received the video-feedback intervention based on the SPIRE happiness model, whereas those in the control group continued their usual family routines and received no study intervention during the trial. Both groups completed the same assessments at T1, T2, and T3. To ensure equitable access, families in the control group were offered the same intervention after all follow-up assessments had been completed.

### 3.2 Sample size

Because this was a pilot randomized controlled trial, the target sample size was determined with reference to both an *a priori* power analysis and sample-size recommendations from previous pilot family-based intervention studies (Whitehead et al., 2016; Saei et al., 2025). An *a priori* power analysis was conducted using G*Power version 3.1.9.7. The calculation was based on the group × time interaction in a repeated-measures analysis of variance. Assuming a medium effect size (*f* = 0.25), an alpha level of 0.05, and statistical power of 0.80, the analysis indicated that at least 24 participants were required to complete the study. Previous group-based parenting interventions involving disadvantaged or vulnerable families have reported attrition rates of approximately 20% (Yang et al., 2024; Law et al., 2025; Hudock et al., 2026). Given that parents in migrant families might also withdraw because of changes in employment, work schedules, or residence, the recruitment target was set at 32 participants.

### 3.3 Participants

Participants were recruited in September 2025 in Nantong, Jiangsu Province, China. Given the pilot nature of the study, convenience sampling was used. Recruitment was conducted through partner kindergartens within the Nantong migrant-child sampling frame, with study information distributed through the kindergartens’ parent committees. The research team screened interested parents according to prespecified inclusion and exclusion criteria. Participants were eligible if: (1) their child was aged 3–6 years, held rural household registration, had moved with the family to an urban area, and had lived there continuously for more than 6 months; (2) they were the child’s mother or father and primary caregiver; (3) they provided informed consent and were able to attend all intervention sessions and follow-up assessments; and (4) they had sufficient communication ability to understand the study procedures and complete the required assessments. Participants were excluded if: (1) they were currently participating in another parent–child interaction or family-based parenting intervention; (2) self-reported medical history or the recruitment interview indicated severe mental illness, such as schizophrenia or bipolar disorder, or severe cognitive impairment that would prevent them from understanding the study, completing the assessments, or participating in the intervention; or (3) work demands, working hours, or other practical circumstances were expected to prevent completion of the intervention sessions or follow-up assessments.

A total of 32 eligible parents of preschool migrant children were recruited. Five withdrew before the baseline assessment because of temporary job transfers, changes in their child’s kindergarten, or other practical circumstances. The remaining 27 participants were randomized, with 14 allocated to the intervention group and 13 to the control group. All randomized participants completed the T1, T2, and T3 assessments and were included in the final analysis.

### 3.4 Intervention

A video-feedback intervention based on the SPIRE happiness model was developed for this study. VFI served as the core delivery technique, and the five SPIRE dimensions were integrated throughout the intervention. The program combined video feedback on parent–child interactions, reflection on parents’ psychological resources, group support, and home practice. It enabled parents to observe their interactions with their children, reflect on their parenting behaviors, and recognize existing personal and relational strengths. Families of preschool migrant children in China may have limited access to social support, opportunities to exchange parenting experiences, and family education resources. To address these constraints and accommodate the cultural context of family education and group-based support in China, the intervention incorporated group games, facilitated discussions, and practical skills training (Park and Hinsz, 2015). Peer exchange and mutual support were used to encourage parental engagement and facilitate implementation.

All sessions were delivered by researchers who had completed 2 weeks of structured training. The training covered the theoretical foundations of the SPIRE happiness model, video-feedback techniques, intervention procedures, and KIPS administration and scoring. Before delivering the intervention, the researchers completed theoretical instruction, simulated practice, and competency assessment. Kindergarten teachers served as co-facilitators and assisted with session organization and communication with parents. Each session was audio-recorded to support the assessment of intervention fidelity. Two external experts with professional qualifications in education and psychology and extensive experience in video-feedback interventions for early family education evaluated adherence to the intervention protocol.

The intervention was delivered in a group format once a week for 6 consecutive weeks. Each session lasted 90 min and was held in the activity center of the partner kindergarten. Each session comprised five components: (1) Theme introduction and group engagement: parents shared experiences and participated in discussions and games to establish a supportive group atmosphere; (2) Video feedback and interaction awareness: recordings of parent–child interactions were used to help parents recognize children’s behavioral cues and identify their own positive interaction behaviors; (3) Skill learning and behavioral rehearsal: practical exercises and role-play activities were used to introduce and consolidate strategies for positive responding, empathic communication, and conflict management; (4) SPIRE-guided reflection and group sharing: parents were encouraged to interpret and discuss parent–child interactions from the perspectives of meaning, emotion regulation, and relational support; and (5) Home practice and continued application: parenting assignments, practical tip sheets, and feedback on home practice were used to support the application of intervention strategies in everyday family situations. Each session therefore followed a sequence of observation, learning, reflection, internalization, and transfer (Tsai and Lee, 2006).

The intervention content was organized around the five dimensions of the SPIRE happiness model (Table 1). Week 1 focused on Intellectual wellbeing and helped parents understand children’s developmental characteristics and improve their ability to observe children’s behavior. Week 2 addressed Spiritual wellbeing and aimed to strengthen parents’ sense of meaning in parenting and positive parenting beliefs. Week 3 focused on Emotional wellbeing and sought to enhance emotional awareness and empathic responsiveness. Week 4 addressed Physical wellbeing, emphasizing stress regulation and positive parent–child engagement. Week 5 focused on Relational wellbeing and aimed to improve listening, communication, and parent–child connectedness. Week 6 integrated all five dimensions and guided parents in reviewing their intervention experiences and developing an individualized plan for sustaining positive parenting practices at home.

**Table 1:** Integrated video-feedback intervention program based on the SPIRE happiness model.

Week	Session theme	Core objectives	Main activities
1	Intellectual wellbeing (I)	Build evidence-informed parenting knowledge and improve understanding of children’s behavior and developmental characteristics.	Welcome participants and establish trust within the group; review clips showing typical child behavioral cues and explain children’s developmental characteristics; practice recognizing child behavioral cues; reflect on and discuss parenting beliefs; complete home practice.
2	Spiritual wellbeing (S)	Strengthen positive parenting beliefs and enhance a sense of meaning in parenting and positive interaction experiences.	Share positive parenting experiences; review strengths in parent–child interactions; practice positive reinforcement strategies; reflect on and discuss positive parenting beliefs; complete home practice.
3	Emotional wellbeing (E)	Enhance emotional awareness and empathic responsiveness and improve emotional interactions between parents and children.	Share parent–child emotional experiences; review clips of emotional interactions and demonstrate emotion-recognition and empathy strategies; practice emotional responding strategies; reflect on and discuss emotion regulation; complete home practice.
4	Physical wellbeing (P)	Identify parenting stress, learn positive self-regulation and conflict-management strategies, and establish stable patterns of supportive physical interaction.	Share parent–child conflict experiences; review clips showing conflict resolution and demonstrate positive forms of physical interaction, including supportive presence, soothing, and physical guidance; practice non-confrontational conflict-resolution strategies; reflect on and discuss responses to conflict; complete home practice.
5	Relational wellbeing (R)	Improve the quality of parent–child communication and strengthen positive interaction and relational connectedness.	Share positive communication experiences; review clips of sensitive responding and identify practical elements of active listening and timely responses; rehearse positive interaction sequences; reflect on and discuss relational support; complete home practice.
6	Integration of all five dimensions	Consolidate positive parenting skills, support the continued application of intervention gains, bring the intervention to a positive close, and maintain long-term mutual support within the group.	Review experiences across the five intervention dimensions and share changes in parent–child interactions; develop individualized parenting plans; encourage continued mutual support among group members; exchange good wishes and conclude the program warmly.

VFI was the central delivery technique. To standardize the procedure, parent–child interactions were recorded for 15 min, consistent with recommendations by Biringen and Easterbrooks (2012) and the 15–20-min observation period recommended for the KIPS (Comfort et al., 2011). The recording included three standardized interaction tasks: free play for 8 min, toy clean-up for 3 min, and shared picture-book reading for 4 min (Biringen and Easterbrooks, 2012; Lawler et al., 2017). After each recording, the research team coded and reviewed the intervention-group videos in relation to the intervention objectives. Three or four representative interaction clips, each lasting 10–20 s, were selected from each recording for use during the feedback sessions (Kennedy et al., 2011). Priority was given to episodes involving parent–child physical contact, sensitive parental responses, and positive reciprocal interaction sequences (Vilaseca et al., 2025). During video feedback, the researchers followed established VFI procedures and used open-ended questioning, positive reinforcement, and guided reflection (Juffer and Bakermans-Kranenburg, 2018; Brookman et al., 2025; Montirosso et al., 2025). These techniques helped parents recognize positive interaction behaviors and better understand their children’s needs. The five SPIRE dimensions were then used to guide reflection on the emotional experiences, parenting values, and relational meanings underlying the observed interactions. This process encouraged parents to move beyond merely observing interactions toward understanding their significance, thereby supporting adjustments in parenting behavior and the mobilization of positive psychological resources.

### 3.5 Outcome measures

#### 3.5.1 Feasibility and acceptability

The assessment of feasibility and acceptability was informed by previous family-based parenting intervention studies that evaluated attendance, retention, participant experience, and adherence to home practice (Klein et al., 2021; Qu et al., 2022). The indicators were adapted to the characteristics of the present intervention. Consistent with previous research, satisfaction and perceived benefits were summarized using participant counts and endorsement rates (Law et al., 2025). The questionnaire was finalized after content review by two experts with experience in family-based interventions for preschool children. Three sets of indicators were assessed:

1.Engagement and Retention Rate: Attendance rate of parents at each intervention session and the number of participants who completed the entire study procedure (T1, T2, and T3).
2.Intervention satisfaction and perceived benefits: Intervention-group parents’ satisfaction with the sessions and activities and their perceptions of the benefits received.
3.Home Practice Compliance: Number of intervention group parents completing weekly parenting strategy practice.

#### 3.5.2 Parent–child interaction behaviors

Parent–child interaction behaviors were assessed using the Keys to Interactive Parenting Scale (KIPS), developed by Comfort and Gordon (2006). Based on a positive parenting framework, the KIPS assesses the quality of interactions between primary caregivers and children aged 2–71 months (Comfort and Gordon, 2006). The scale included three dimensions: Building Relationships, Promoting Learning, and Supporting Confidence, with a total of 12 items. Each item is rated by a trained observer on a 5-point rating scale based on a 15–20-min recording of parent–child interaction. Higher total scores indicate more positive caregiver interaction behaviors. Previous research has supported the reliability, validity, and applicability of the KIPS across families from different cultural and socioeconomic backgrounds (Comfort et al., 2011; Cañas et al., 2020). Both raters in the present study completed standardized training and were qualified to administer and score the KIPS. They independently rated all 27 parent–child interaction videos collected at T1. Interrater reliability was high, ICC (2.1) = 0.916.

#### 3.5.3 Family parenting environment

The quality of the child’s family parenting environment was assessed using the Family Parenting Environment Scale for Children Aged 3–6 Years, developed by He (2008). The scale was developed with reference to established measures, including the Egna Minnen av. Barndoms Uppfostran questionnaire (EMBU) and the Home Observation for Measurement of the Environment Inventory (HOME Inventory; Perris et al., 1980; Caldwell and Bradley, 1978), and was designed for families of preschool children in China. Previous research has supported its applicability in Chinese family contexts (He et al., 2009; Lan and Li, 2020). The scale includes six dimensions with 53 items: Emotional Warmth/Self-Expression, Neglect/Interference/Punishment, Language/ Cognition, Social Adaptation/Self-Management, Environmental Atmosphere, and Activity Diversity/ Game Participation. A 5-point Likert scale (1 = never, 5 = always) was used, with items in the Neglect/Interference/Punishment dimension reverse-scored. Higher total scores indicated a higher quality of the child’s family parenting environment. The participating parent completed the scale at T1, T2, and T3. In this study, Cronbach’s α for the total scale was 0.890, and for subscales ranged from 0.703–0.836.

#### 3.5.4 Parenting sense of competence

The Parenting Sense of Competence Scale (PSOC) developed by Johnston and Mash (1989) was used to assess parenting efficacy. The Chinese version has demonstrated good reliability and validity among Chinese parents of preschool children (Li et al., 2021). The scale includes two dimensions: Satisfaction and Self-Efficacy, with a total of 17 items. A 6-point Likert scale (1 = strongly disagree, 6 = strongly agree) was used, and the satisfaction dimension items were reverse-scored. Higher total scores indicated a stronger sense of parenting competence. The participating parent completed the PSOC at T1, T2, and T3. In this study, Cronbach’s α for the total scale, Satisfaction, and Self-Efficacy were 0.783, 0.785, and 0.760, respectively.

#### 3.5.5 Perceived social support

The Chinese version of the Perceived Social Support Scale (PSSS) developed by Zimet et al. (1988) and revised by Jiang (2001) was used to measure parents’ subjective perceptions of social support. The scale has been validated in Chinese parent samples (Xu and Tang, 2023; Yue et al., 2022). The scale includes three dimensions: Family Support, Friend Support, and Significant Other Support, with a total of 12 items. A 7-point Likert scale (1 = very strongly disagree, 7 = very strongly agree) was used. Higher total scores indicated higher levels of perceived social support. The participating parent completed the PSSS at T1, T2, and T3. In this study, Cronbach’s α for the total scale was 0.878, and for the subscales ranged from 0.811–0.864.

#### 3.5.6 Child emotional and behavioral problems

Child emotional and behavioral problems were assessed using the Strengths and Difficulties Questionnaire (SDQ; Goodman, 2001) as an exploratory child-level outcome. The SDQ has demonstrated satisfactory reliability and validity for identifying emotional and behavioral problems among preschool children in China (Du et al., 2008). The 20 items from the Emotional Symptoms, Conduct Problems, Hyperactivity, and Peer Problems subscales were used to calculate the Total Difficulties score. A 3-point Likert scale (0 = not true, 1 = somewhat true, and 2 = certainly true) was used. Higher total scores indicated more prominent emotional and behavioral problems in children. The participating parent completed the SDQ at T1, T2, and T3. In this study, Cronbach’s α for the total scale was 0.764, and for the subscales ranged from 0.731 to 0.772.

### 3.6 Statistical analysis

All quantitative analyses were conducted using IBM SPSS Statistics version 25.0. Frequencies and percentages were used to summarize categorical variables, whereas means and standard deviations were used for continuous variables. Baseline sociodemographic characteristics and outcome scores were compared between the intervention and control groups to examine baseline differences. Independent-samples t tests were used for continuous variables, and chi-square tests or Fisher’s exact tests were used for categorical variables, as appropriate. All tests were two-tailed. The normality of the outcome variables was assessed using the Shapiro–Wilk test. For normally distributed outcomes, repeated-measures analyses of variance were conducted with group as the between-subjects factor and assessment time as the within-subjects factor. Outcomes were compared across T1, T2, and T3, with the group × time interaction representing the primary effect of interest. Partial eta squared (*η_p_^2^*) was used as the effect-size measure. Following Cohen (1988) criteria, values of 0.01, 0.06, and 0.14 were interpreted as small, medium, and large effects, respectively. When a significant group × time interaction was identified, simple-effects analyses were conducted. Pairwise comparisons were performed using the least significant difference procedure to examine changes from T1 to T2 and from T1 to T3, as well as between-group differences at corresponding assessment points. A Bonferroni-adjusted significance threshold of *p* < 0.0167 was applied to these comparisons. For all other statistical tests, significance was set at *p* < 0.05. All 27 randomized participants completed the assessments at T1, T2, and T3. Therefore, there were no post-randomization missing data, and no missing-data imputation was performed.

## 4 Results

### 4.1 Baseline comparisons

#### 4.1.1 Sociodemographic characteristics

A total of 27 participants completed the study procedure: 14 in the intervention group and 13 in the control group. The sociodemographic characteristics of the two groups are presented in Table 2. Both groups were predominantly mothers (*n* = 24, 88.9%) with only three fathers (two in the intervention group and one in the control group). The participants were mainly aged 31–40 years (*n* = 20, 74.1%), and most were married (*n* = 24, 88.9%). Nearly two-thirds of the participants had a high school education or less (*n* = 17, 63.0%), and the vast majority were employed full- or part-time (*n* = 23, 85.2%). More than half of the participants had a family income between RMB 50, 000 and 150, 000 (*n* = 17, 63.0%). Chi-square test results exhibited no significant differences between the two groups in any sociodemographic characteristic (*p* > 0.05).

**Table 2:** Baseline sociodemographic characteristics of participants.

Category		Intervention group n(%)	Control group n(%)	*χ^2^*	*p*
Caregiver role	Father	2(14.3)	1(7.7)	0.297	0.586
	Mother	12(85.7)	12(92.3)		
Age (years)	≤30	2(14.3)	3(23.1)	0.671^*^	0.816
	31–40	11(78.6)	9(69.2)		
	≥41	1(7.1)	1(7.7)		
Marital status	Married	13(92.9)	11(84.6)	2.692^*^	0.347
	Divorced	0(0.0)	2(15.4)		
	Widowed	1(7.1)	0(0.0)		
Education level	High school or below	10(71.4)	7(53.8)	0.894	0.345
	College and above	4(28.6)	6(46.2)		
Employment	Homemaker/unemployed	3(21.4)	1(7.7)	1.083^*^	0.834
	Full-time	9(64.3)	10(76.9)		
	Part-time	2(14.3)	2(15.4)		
Family income	<50,000 RMB	4(28.6)	3(23.1)	3.309^*^	0.415
	50,001–100,000 RMB	7(50.0)	3(23.1)		
	100,001–150,000 RMB	2(14.3)	5(38.5)		
	>150,000 RMB	1(7.1)	2(15.4)		

^*^Fisher’s exact test.

#### 4.1.2 Baseline outcome measures

Independent samples *t*-test results (Table 3) exhibited no significant differences between the intervention and control groups in baseline scores for parent–child interaction behaviors, the family parenting environment, parenting sense of competence, perceived social support, and child emotional and behavioral problems (*p* > 0.05).

**Table 3:** Baseline outcome measures.

Variable	Intervention Group (*n* = 14) M ± SD	Control group (*n* = 13) M ± SD	*t*	*p*
Building relationships	14.82 ± 2.45	14.46 ± 2.76	0.359	0.723
Promoting learning	11.61 ± 1.58	11.00 ± 1.47	1.030	0.312
Supporting confidence	8.43 ± 1.27	7.62 ± 1.49	1.532	0.141
Parent–child interaction total	34.85 ± 4.73	33.12 ± 5.05	0.925	0.364
Emotional warmth/self-expression	30.64 ± 4.47	32.00 ± 3.83	−0.844	0.404
Neglect/interference/punishment	39.93 ± 2.57	39.46 ± 7.30	−0.154	0.883
Language/cognition	45.43 ± 3.67	46.77 ± 3.22	−1.005	0.322
Social adaptation/self-management	41.00 ± 4.87	42.31 ± 3.71	−0.781	0.442
Environmental atmosphere	19.14 ± 2.51	20.08 ± 1.50	−1.163	0.256
Activity diversity/game participation	23.86 ± 3.35	23.69 ± 3.66	0.122	0.904
Family parenting environment total	199.21 ± 15.04	204.31 ± 14.95	−0.882	0.386
Satisfaction	33.79 ± 4.61	33.77 ± 8.26	0.006	0.995
Self-efficacy	32.00 ± 4.88	30.69 ± 5.28	0.669	0.510
Parenting sense of competence total	65.79 ± 6.03	64.46 ± 11.59	0.377	0.710
Family support	23.00 ± 2.75	22.38 ± 3.04	0.552	0.586
Friend support	20.14 ± 3.84	21.23 ± 2.89	−0.826	0.416
Significant other support	20.71 ± 3.07	20.92 ± 2.96	−0.180	0.859
Perceived social support total	63.86 ± 8.49	64.54 ± 7.50	−0.220	0.827
Emotional symptoms	2.50 ± 1.22	2.54 ± 1.98	−0.061	0.952
Conduct problems	2.21 ± 1.48	2.15 ± 1.41	0.109	0.914
Hyperactivity	4.14 ± 1.88	4.38 ± 1.26	−0.390	0.700
Peer relationship problems	5.21 ± 1.48	4.85 ± 1.34	0.676	0.506
Emotional/behavioral problems total	14.07 ± 4.76	13.92 ± 4.86	0.080	0.937

### 4.2 Study outcomes

#### 4.2.1 Feasibility and acceptability

All 27 randomized participants completed the study and all three assessments, resulting in a retention rate of 100%. No participants withdrew after randomization. Among parents in the intervention group, attendance was 100% across all six sessions, suggesting that the intervention was feasible to implement among families of preschool migrant children. Fourteen parents in the intervention group were invited to provide feedback on the activity evaluation. Among them, 13 expressed satisfaction with the overall effectiveness of the activity (92.9%); 13 (92.9%) reported benefits in emotional support, parenting confidence, and optimizing parent–child interaction bonds; and 14 (100%) reported gaining peer support. Twelve parents (85.8%) indicated that they would actively practice the learned parenting methods in daily parenting, and would be willing to continue participating in similar VFIs in the future (Table 4). These findings suggested a high level of acceptability among participating parents (Table 4).

**Table 4:** Satisfaction with and perceived benefits of the video-feedback intervention among the intervention group (*n* = 14).

Course and activity evaluation	*n*	Selection rate (%)	Perceived benefits	*n*	Selection rate (%)
Overall satisfaction	13	92.9	Gained emotional support and parenting confidence	13	92.9
Satisfaction with theme design	13	92.9	Optimized parent–child interaction bond	13	92.9
Satisfaction with activity format	14	100	Group peer support and experience sharing	14	100
Satisfaction with facilitator performance	14	100	Actively practiced parenting methods weekly	12	85.8
Comfortable and safe activity atmosphere	13	92.9	Willing to continue participating in similar interventions	12	85.8
Appropriate difficulty, easy to apply	12	85.8			

#### 4.2.2 Parent–child interaction behaviors

As shown in Table 5, significant group × time interactions were observed for Building Relationships, Promoting Learning, Supporting Confidence, and the total parent–child interaction score, with *F* values ranging from 20.14 to 28.07, all *p* < 0.001, and partial η^2^ values ranging from 0.446 to 0.529. According to Cohen’s criteria, all partial η^2^ values were in the large range. Simple-effects analyses showed that, within the intervention group, scores on all three outcomes were significantly higher at T2 and T3 than at T1. Their scores were also significantly higher than those of the control group at the corresponding assessment points, based on the Bonferroni-adjusted threshold of *p* < 0.0167. No statistically significant changes over time were observed in the control group. For the total parent–child interaction score, the between-group mean difference was 17.80 at T2, 95% CI (13.72, 21.88), and 17.68 at T3, 95% CI (13.75, 21.62). This above results indicates that the VFI based on the SPIRE happiness model has a preliminary intervention effect on improving parent–child interaction behaviors. Mean differences and 95% confidence intervals for all outcomes at each assessment point are presented in Supplementary Table S1.

**Table 5:** Parent–child interaction outcomes across three assessment points.

Variable		Group	*n*	Pre-intervention (T1) M ± SD	Post-intervention (T2) M ± SD	3-month follow-up (T3) M ± SD	Time × group F	η_p_^2^
Parent–child interaction	Parent–child interaction	Intervention	14	14.82 ± 2.45	22.00 ± 3.29^ab^	22.68 ± 2.25^ab^	20.60^***^	0.452
		Control	13	14.46 ± 2.76	14.58 ± 1.53	15.62 ± 2.17		
	Promoting learning	Intervention	14	11.61 ± 1.58	17.32 ± 1.66^ab^	17.32 ± 1.85^ab^	23.53^***^	0.510
		Control	13	11.00 ± 1.47	11.65 ± 1.18	11.77 ± 1.28		
	Supporting confidence	Intervention	14	8.43 ± 1.27	12.75 ± 1.79^ab^	13.11 ± 1.51^ab^	20.14^***^	0.446
		Control	13	7.62 ± 1.49	7.73 ± 1.33	8.04 ± 1.64		
	Total score	Intervention	14	34.86 ± 4.73	52.07 ± 6.16^ab^	53.11 ± 5.23^ab^	28.07^***^	0.529
		Control	13	33.12 ± 5.05	33.96 ± 3.53	35.42 ± 4.65		
		Control	13	13.92 ± 4.86	11.69 ± 3.86	11.54 ± 3.91		

Within the same group, ^a^significant difference compared to pre-intervention; at the same time point, ^b^significant difference compared to the control group (LSD test, Bonferroni-corrected *p* < 0.0167). ^*^p < 0.05, ^**^p < 0.01, ^***^p < 0.001 (for F-test).

#### 4.2.3 Family parenting environment

Significant group × time interactions were observed for Emotional Warmth/Self-Expression, Language/Cognition, Social Adaptation/Self-Management, Environmental Atmosphere, and the total family parenting environment score (Table 6), with *F* values ranging from 4.85 to 9.50, *p* < 0.05, and partial *η^2^* values ranging from 0.162 to 0.275. According to Cohen’s criteria, all effect-size estimates were in the large range. Simple-effects analyses showed that, within the intervention group, scores on these outcomes were significantly higher at T2 and T3 than at T1. At T3, the intervention group also scored significantly higher than the control group on these outcomes, based on the Bonferroni-adjusted threshold of *p* < 0.0167. No statistically significant changes over time were observed in the control group. For the total family parenting environment score, the between-group mean difference was 21.65 at T2, 95% CI (10.17, 33.12), and 33.87 at T3, 95% CI (18.23, 49.51). The above results suggesting that the VFI based on the SPIRE happiness model has a preliminary intervention effect on improving the family parenting environment. The group × time interactions were not statistically significant for Neglect/Interference/Punishment or Activity Diversity/Game Participation (both *p* > 0.05). Mean differences and 95% confidence intervals for all assessment points are reported in Supplementary Table S1.

**Table 6:** Secondary outcomes across three assessment points.

Variable		Group	*n*	Pre-intervention (T1) M ± SD	Post-intervention (T2) M ± SD	3-month follow-up (T3) M ± SD	Time × group F	η_p_^2^
Family parenting environment	Emotional warmth	Intervention	14	30.64 ± 4.47	37.71 ± 2.46^ab^	39.21 ± 1.37^ab^	9.50^***^	0.275
		Control	13	32.00 ± 3.83	34.38 ± 3.78	31.85 ± 3.91		
	Neglect/interference/punishment	Intervention	14	39.14 ± 2.57	47.00 ± 4.04	43.07 ± 5.37	1.03	0.04
		Control	13	39.46 ± 7.30	43.54 ± 5.81	39.77 ± 6.30		
	Language/cognition	Intervention	14	45.43 ± 3.67	55.36 ± 4.60^ab^	55.86 ± 4.31^ab^	8.36^**^	0.251
		Control	13	46.77 ± 3.22	48.85 ± 4.20	46.92 ± 7.12		
	Social adaptation/self-management	Intervention	14	41.00 ± 4.87	49.21 ± 5.37^ab^	49.93 ± 5.23^ab^	4.85^*^	0.162
		Control	13	42.31 ± 3.71	41.92 ± 5.44	43.38 ± 6.44		
	Environmental atmosphere	Intervention	14	19.14 ± 2.51	21.79 ± 2.89^a^	23.50 ± 1.87^ab^	6.03^**^	0.194
		Control	13	20.08 ± 1.50	20.85 ± 1.46	19.77 ± 2.98		
	Activity diversity/game participation	Intervention	14	23.86 ± 3.35	26.50 ± 3.32	27.14 ± 2.66	3.01	0.107
		Control	13	23.69 ± 3.66	26.38 ± 2.79	23.15 ± 3.72		
	Total score	Intervention	14	199.21 ± 15.04	237.57 ± 16.64^ab^	238.71 ± 15.79^ab^	7.63^**^	0.234
		Control	13	204.31 ± 14.95	215.92 ± 11.67	204.85 ± 23.23		
Parenting sense of competence	Satisfaction	Intervention	14	33.79 ± 4.61	38.00 ± 3.11^ab^	40.36 ± 4.80^ab^	4.44^*^	0.151
		Control	13	33.77 ± 8.26	32.92 ± 5.02	32.77 ± 4.34		
	Self-efficacy	Intervention	14	32.00 ± 4.88	36.50 ± 3.44^ab^	38.86 ± 3.98^ab^	10.92^**^	0.304
		Control	13	30.69 ± 5.28	30.38 ± 5.77	28.77 ± 2.83		
	Total score	Intervention	14	65.79 ± 6.03	74.50 ± 5.27^ab^	79.21 ± 8.30^ab^	11.10^**^	0.307
		Control	13	64.46 ± 11.59	63.31 ± 6.94	61.54 ± 5.21		
Perceived social support	Family Support	Intervention	14	23.00 ± 2.75	26.43 ± 1.74^ab^	27.43 ± 1.16^ab^	5.23^**^	0.154
		Control	13	22.38 ± 3.04	22.62 ± 3.23	22.15 ± 3.21		
	Friend support	Intervention	14	20.14 ± 3.84	22.43 ± 4.67	24.50 ± 2.35^ab^	3.22^*^	0.114
		Control	13	21.23 ± 2.89	21.46 ± 3.82	20.77 ± 3.75		
	Significant other support	Intervention	14	20.71 ± 3.07	24.50 ± 2.24^ab^	25.86 ± 2.54^ab^	5.12^**^	0.153
		Control	13	20.92 ± 2.96	21.38 ± 3.82	20.54 ± 3.64		
	Total score	Intervention	14	63.86 ± 8.49	73.36 ± 7.56^ab^	77.79 ± 5.29^ab^	5.35^**^	0.162
		Control	13	64.54 ± 7.50	65.46 ± 10.30	63.46 ± 10.15		
Child emotional and behavioral Problems	Emotional symptoms	Intervention	14	2.50 ± 1.22	2.07 ± 1.59	1.14 ± 1.10	1.51	0.057
		Control	13	2.54 ± 1.98	2.23 ± 1.54	2.31 ± 1.70		
	Conduct Problems	Intervention	14	2.21 ± 1.48	1.71 ± 0.91	1.86 ± 0.53	0.15	0.006
		Control	13	2.15 ± 1.41	1.62 ± 1.12	1.54 ± 0.88		
	Hyperactivity	Intervention	14	4.14 ± 1.88	3.21 ± 1.19	3.29 ± 1.33	0.03	0.001
		Control	13	4.38 ± 1.26	3.54 ± 1.27	3.69 ± 1.44		
	Peer relationship problems	Intervention	14	5.21 ± 1.48	4.00 ± 1.24	3.86 ± 0.77	0.52	0.020
		Control	13	4.85 ± 1.34	4.31 ± 1.32	4.00 ± 1.35		
	Total score	Intervention	14	14.07 ± 4.76	11.00 ± 2.86	10.14 ± 2.51	0.34	0.013
		Control	13	13.92 ± 4.86	11.69 ± 3.86	11.54 ± 3.91		

Within the same group, ^a^significant difference compared to pre-intervention; at the same time point, ^b^significant difference compared to the control group (LSD test, Bonferroni-corrected *p* < 0.0167). ^*^p < 0.05, ^**^p < 0.01, ^***^p < 0.001 (for F-test).

#### 4.2.4 Parenting sense of competence

Significant group × time interactions were observed for Satisfaction, Self-Efficacy, and the total Parenting Sense of Competence Scale score (Table 6), with *F* values ranging from 4.44 to 11.10, all *p* < 0.05, and partial η^2^ values ranging from 0.151 to 0.307. According to Cohen’s criteria, all partial η^2^ values were in the large range. Simple-effects analyses showed that, within the intervention group, scores on all three outcomes were significantly higher at T2 and T3 than at T1. At both T2 and T3, the intervention group also scored significantly higher than the control group on all three outcomes, based on the Bonferroni-adjusted significance threshold of *p* < 0.0167. No statistically significant changes over time were observed in the control group. For the total parenting sense of competence score, the between-group mean difference was 11.19 at T2, 95% CI (6.33, 16.06), and 17.68 at T3, 95% CI (12.13, 23.22). The above results suggest that the VFI based on the SPIRE happiness model has a preliminary intervention effect on enhancing parents’ sense of parenting efficacy. Mean differences and 95% confidence intervals for all assessment points are reported in Supplementary Table S1.

#### 4.2.5 Perceived social support

Significant group × time interactions were observed for Family Support, Friend Support, Significant Other Support, and the total perceived social support score (Table 6), with *F* values ranging from 3.22 to 5.35, all *p* < 0.05, and partial η^2^ values ranging from 0.114 to 0.176. According to Cohen’s criteria, the partial η^2^ values ranged from medium to large. Simple-effects analyses showed that, within the intervention group, Family Support, Significant Other Support, and total perceived social support scores were significantly higher at T2 than at T1. At T3, scores on all four perceived social support outcomes were significantly higher than at T1 and significantly higher than those of the control group, based on the Bonferroni-adjusted significance threshold of *p* < 0.0167. No statistically significant changes over time were observed in the control group. For the total perceived social support score, the between-group mean difference was 7.90 at T2, 95% CI (0.77, 15.02), and 14.32 at T3, 95% CI (7.98, 20.67). The estimated between-group difference was numerically larger at T3 than at T2. The above results suggest that the VFI based on the SPIRE happiness model has a preliminary intervention effect in enhancing parents’ understanding of social support. Mean differences and 95% confidence intervals for all assessment points are reported in Supplementary Table S1.

#### 4.2.6 Child emotional and behavioral problems

No statistically significant group × time interactions were observed for Emotional Symptoms, Conduct Problems, Hyperactivity, Peer Problems, or the total emotional/behavioral problems scores (Table 6) (all *p* > 0.05). For the SDQ Total Difficulties score, the intervention group had a lower mean score than the control group at T2, with a between-group mean difference of −0.69, 95% CI (−3.37, 1.99), and at T3, with a mean difference of −1.40, 95% CI (−3.98, 1.19). Although the point estimate indicated a larger numerical difference at T3, the 95% confidence intervals at both assessment points included zero, and neither between-group difference was statistically significant. This result merely presents a preliminary trend that is favorable for intervention. It does not yet have statistical evidence to support it. Further studies with larger sample sizes and longer follow-up periods are needed to explore the potential effects of the intervention on children’s emotional and behavioral problems. Mean differences and 95% confidence intervals for all assessment points are reported in Supplementary Table S1.

## 5 Discussion

This pilot randomized controlled trial examined the feasibility, acceptability, and preliminary outcomes of a video-feedback intervention informed by the SPIRE happiness model of happiness among families with preschool migrant children in China. The intervention appeared feasible and acceptable within the present sample, as indicated by complete attendance and retention, high adherence to home practice, high satisfaction, and positive perceived-benefit ratings. Relative to the control group, participants in the intervention group showed greater improvements in parent–child interaction behaviors, the family parenting environment, parenting sense of competence, and perceived social support. Between-group differences in these outcomes were also observed at the 3-month follow-up. By contrast, no statistically significant between-group differences were found in child emotional and behavioral problems. These findings provide preliminary outcome estimates and implementation information that may inform a larger, adequately powered trial.

In terms of feasibility and acceptability, this intervention has a relatively high level of feasibility and acceptance among Chinese families of preschool migrant children. This might be related to the design of the intervention. First, delivering the sessions in a kindergarten was convenient for parents’ daily routines and reduced the time and travel burden associated with participation. Second, the video-feedback component used recordings of parents’ own interactions with their children, making the intervention content directly relevant to everyday parenting situations. This contextual relevance may have helped parents understand the feedback and increased their willingness to apply the strategies at home (Wan et al., 2025). Third, the group format and emphasis on positive psychological resources created opportunities for parents to exchange parenting experiences and receive emotional and peer support. Similar benefits of group-based family support have been reported in interventions involving disadvantaged families (McNaughton et al., 2014; Vilaseca et al., 2025). Nevertheless, the feasibility and acceptability findings reflect a small and potentially highly motivated sample from one setting. The intervention’s acceptability and practical applicability therefore require further evaluation across regions and among migrant families with more diverse circumstances.

The greater improvements in parent–child interaction behaviors observed in the intervention group are consistent with previous VFI research reporting increases in parental sensitivity, responsiveness, and positive interaction behaviors (Euser et al., 2021). VFI draws parents’ attention to concrete episodes within recorded interactions, helping them identify children’s behavioral cues and recognize responsive behaviors that are already present in their parenting (O’Hara et al., 2019; van IJzendoorn et al., 2023). Repeated observation and guided reflection may make positive interaction patterns more visible and support their application in subsequent parent–child interactions. The present program extended this conventional VFI approach by integrating the SPIRE happiness model into the feedback and reflection process. Rather than focusing only on interaction techniques, the program also directed parents’ attention to emotional experiences, meaning in parenting, personal strengths, and relational connectedness. This broader emphasis may be particularly relevant for families of preschool migrant children, who may experience financial pressure, time constraints, and limited access to parenting resources. Under such conditions, the use of positive interaction strategies may depend not only on knowledge and communication skills but also on parents’ emotional functioning, sense of meaning, and experiences within family relationships (Crandall et al., 2015; Silvers et al., 2017; Fischer et al., 2024; Ismair et al., 2025). The Intellectual, Emotional, and Relational dimensions of the SPIRE happiness model may have helped parents interpret children’s behavior more constructively, reflect on their own emotional responses, and recognize the value of positive relational experiences. The observed changes in parent–child interaction may therefore have been associated with both the behavioral feedback provided by VFI and the broader reflection encouraged by the SPIRE framework. However, because the study did not test mediating processes, the contribution of these psychological and relational factors remains tentative.

Improvements were also observed in four dimensions of the family parenting environment: Emotional Warmth/Self-Expression, Language/Cognition, Social Adaptation/Self-Management, and Environmental Atmosphere. These findings are consistent with the close and potentially reciprocal relationship between everyday parent–child interactions and the broader home environment. Parents may adjust their communication, emotional responses, and learning support through repeated interactions with their children, and these recurring practices may gradually become features of the family environment. Conversely, a warm, supportive, and cognitively stimulating home environment may facilitate positive parent–child interaction (Cooke et al., 2019). The intervention’s attention to meaning in parenting, emotional awareness, and relational support may also have contributed to a more supportive family atmosphere (Hajal and Paley, 2020). Previous research similarly suggests that family parenting environments reflect the combined influence of parenting practices, parental psychological functioning, and family relationships (Chen et al., 2000; Cooke et al., 2019; Thorup et al., 2022). Nevertheless, the present study did not examine the direction of these associations or test mediation and moderation pathways; the proposed explanations should therefore be regarded as hypotheses for future research. No statistically significant changes were found in Activity Diversity/Game Participation or Neglect/Interference/Punishment. One possible explanation is that parents in migrant families may have limited time for varied parent–child activities because of long or inflexible working hours (Condon and McClean, 2017). In addition, the intervention primarily reinforced existing strengths and positive parenting behaviors, while providing less direct training in reducing intrusive, punitive, or otherwise negative parenting practices. Future versions of the program could include brief parent–child play activities that are compatible with families’ time constraints and more targeted components addressing negative parenting behaviors.

Parenting sense of competence also increased in the intervention group relative to the control group. Previous research suggests that VFI may strengthen parents’ perceptions of competence by making successful parenting behaviors visible within actual interactions (O’Hara et al., 2019; Johal et al., 2026). Seeing themselves respond sensitively, communicate effectively, or engage positively with their children may help parents recognize abilities that they had previously overlooked. However, existing VFI research has focused predominantly on observable interaction behaviors, and evidence concerning parenting beliefs and perceived competence remains comparatively limited (Waters et al., 2021; Starreveld et al., 2024). The SPIRE-informed reflection incorporated into the present program may have broadened this process by encouraging parents to consider their personal strengths, parenting values, and sense of meaning. In combination with feedback on specific positive behaviors, this reflection may have supported more positive evaluations of parenting competence. This interpretation is consistent with social cognitive theory, which emphasizes the role of self-efficacy in behavioral choice, effort, and persistence (Bandura, 2024). Parenting self-efficacy has also been associated with more positive parenting practices and higher-quality parent–child interactions (Hill and Bush, 2001; Glatz et al., 2024). Integrating positive psychological resources into VFI may therefore offer a useful perspective for understanding how parents’ beliefs about their competence and their observable parenting behaviors are connected. This proposed process, however, requires direct testing.

The findings for perceived social support suggest that the intervention may also have influenced how parents viewed their available relational resources. Two features of the program may have contributed to this outcome. At the content level, the Relational dimension of the SPIRE happiness model emphasized interpersonal connection, supportive communication, and the value of positive relationships. At the implementation level, the fixed-membership group format provided parents with opportunities to exchange experiences and receive peer encouragement. Group-based family education programs have similarly been associated with stronger perceived social support among disadvantaged families (Barlow et al., 2016; Heubeck et al., 2025). Friend Support did not differ significantly from baseline immediately after the intervention but was higher at the 3-month follow-up. This delayed pattern may indicate that some perceptions of social support develop gradually as parents continue to interact with others and accumulate supportive relational experiences (Burns and Martin, 2019). Nevertheless, the study did not assess whether participants maintained contact after the intervention or identify the sources of perceived support. The interpretation of this delayed change therefore remains provisional. More broadly, these findings suggest that the VFI based on the SPIRE happiness model may be relevant not only to interaction within the parent–child dyad but also to parents’ wider experiences of relational support.

Contrary to the third hypothesis, no statistically significant between-group differences were found in child emotional and behavioral problems. Although the point estimates were in the expected direction, the confidence intervals included zero. This result differs from findings from some parent management training and parent–child therapy programs that reported reductions in child behavioral problems (Doyle et al., 2016; Hudson et al., 2023; Xu et al., 2024). Several features of the present study may help explain the absence of detectable differences. First, the intervention directly targeted parents’ interaction behaviors, psychological resources, and the home parenting context, whereas children’s emotional and behavioral problems represented a more distal outcome. Changes in distal child outcomes may require sustained changes in family processes over a longer period (Yu et al., 2025). Children’s social and emotional functioning is also influenced by ongoing interaction experiences, caregiver psychological functioning, and the home environment (Clarke-Stewart and Dunn, 2006; Quigley et al., 2025). Second, child emotional and behavioral functioning reflects influences extending beyond the immediate family, including genetic characteristics, neighborhood conditions, and peer relationships (Fu and Zuo, 2011; Ramsdell and Brock, 2021; Reiss et al., 2022). A 6-week intervention followed by a 3-month assessment period may not have been sufficient to detect changes in this multifactorial outcome. Children in both groups also attended the same kindergarten and received routine educational support, which may have reduced between-group differentiation (Yuan and Luo, 2026). Larger trials with longer follow-up periods are needed to determine whether changes in parenting and family processes are followed by later changes in child emotional and behavioral functioning.

### 5.1 Limitations

Several limitations should be considered. First, the sample was small and was recruited from a single kindergarten in one geographical area. These features restrict the extent to which the findings can be generalized to migrant families in other regions or educational contexts. Larger, multi-site trials are needed to evaluate both intervention outcomes and implementation across settings. Second, most participants were mothers, with few fathers and no other caregivers represented. Grandparental care and caregiving arrangements involving multiple adults are common in some Chinese migrant families, and caregivers may differ in their parenting needs, family roles, and responses to intervention (Liang and Van Leeuwen, 2025). Future studies should recruit more diverse caregivers and examine whether intervention delivery requires adaptation for different caregiving arrangements. Third, the follow-up period was limited to 3 months. The study therefore cannot establish whether the observed between-group differences would continue over longer periods. Additional follow-up assessments are required to evaluate the longer-term course of the outcomes. Fourth, the program adopted a predominantly strengths-based approach, emphasizing positive parenting behaviors and parents’ psychological resources. It included less direct content addressing negative parenting behaviors and relatively limited support for parent–child activities adapted to the time constraints of migrant families. Future refinement should broaden these components while preserving the intervention’s brief and accessible format. Finally, the intervention combined VFI, the SPIRE happiness model, group activities, and home practice. The present design could not determine the independent contribution of each component. Dismantling designs, factorial studies, or multi-arm randomized trials could help clarify which components are necessary, beneficial, or potentially redundant (Skivington et al., 2021).

## 6 Conclusion

This pilot randomized controlled trial suggests that the VFI based on the SPIRE happiness model was feasible and acceptable among participating Chinese families with preschool migrant children. The intervention was associated with preliminary improvements in parent–child interaction behaviors, the family parenting environment, parenting sense of competence, and perceived social support, whereas no statistically significant differences were found in child emotional and behavioral problems. By integrating video feedback with reflection on positive psychological and relational resources, the program provides a preliminary framework for a family-based intervention tailored to the circumstances of migrant families. A larger, adequately powered trial with broader recruitment and longer follow-up is needed to evaluate the intervention and clarify the processes through which any benefits may occur.

## Data Availability

The original contributions presented in the study are included in the article/Supplementary material, further inquiries can be directed to the corresponding author.
