# Supplementary material for: Effectiveness of a video-feedback intervention based on the SPIRE happiness model for promoting family parenting among preschool migrant children: a pilot randomized controlled trial
Source: Front Psychol. 2026 Sep 11;17:1915668. doi: 10.3389/fpsyg.2026.1915668 (PMC13612549; doi:10.3389/fpsyg.2026.1915668)
Supplement: Supplementary file 1 [file Data_Sheet_1.PDF]

Supplementary Material

Supplementary Table S1. Between-group Mean Differences and 95% Confidence Intervals for Outcome Measures at T2 and T3

Variable	T2 MD (95% CI)	T3 MD (95% CI)
Building Relationships	7.42（5.36,9.48）	7.06（5.31,8.82）
Promoting Learning	5.36（4.14,6.58）	5.55（4.28,6.82）
Supporting Confidence	5.02（3.76,6.28）	5.07（3.82,6.32）
Parent-Child Interaction Total	17.80（13.72,21.88）	17.68（13.75,21.62）
Emotional Warmth/Self-Expression	3.33（0.82,5.84）	7.37（5.08,9.66）
Neglect/Interference/Punishment	3.46（-0.48,7.40）	3.30（-1.33,7.93）
Language/Cognition	6.51（3.01,10.01）	8.93（4.31,13.56）
Social Adaptation/Self-Management	7.29（3.01,11.58）	6.54（1.91,11.18）
Environmental Atmosphere	0.94（-0.90,2.78）	3.73（1.78,5.69）
Activity Diversity/Game Participation	0.12（-2.33,2.56）	3.99（1.44,6.53）
Family Parenting Environment Total	21.65（10.17,33.12）	33.87（18.23,49.51）
Satisfaction	5.08（1.79,8.36）	7.59（3.95,11.23）
Self-Efficacy	6.12（2.39,9.85）	10.09（7.33,12.85）
Parenting Sense of Competence Total	11.19（6.33,16.06）	17.68（12.13,23.22）
Family Support	2.96（0.61,5.30）	5.28（3.39,7.16）
Friend Support	0.97（-2.43,4.37）	3.73（1.27,6.19）
Significant Other Support	1.54（0.10,2.98）	5.32（2.85,7.79）
Perceived Social Support Total	7.90（0.77,15.02）	14.32（7.98,20.67）
Emotional Symptoms	-0.16（-1.40,1.08）	-1.17（-2.29,0.04）
Conduct Problems	0.10（-0.71,0.91）	0.32（-0.25,0.89）
Hyperactivity	-0.32（-1.30,0.65）	-0.41（-1.50,0.69）
Peer Relationship Problems	-0.31（-1.32,0.71）	-0.14（-1.01,0.72）
Emotional/Behavioral Problems Total	-0.69（-3.37,1.99）	-1.40（-3.98,1.19）
